# Does having Turner syndrome affect quality of life in Brazilian women compared to common population?

**DOI:** 10.20945/2359-3997000000136

**Published:** 2019-04-26

**Authors:** Maria Bernarda Estevez, Patricia Teofilo Monteagudo, Kelly Christina Oliveira, Ieda Therezinha do Nascimento Verreschi

**Affiliations:** 1 Universidade Federal de São Paulo Ambulatório de Endocrinologia Escola Paulista de Medicina Universidade Federal de São Paulo São Paulo SP Brasil Ambulatório de Endocrinologia, Escola Paulista de Medicina, Universidade Federal de São Paulo (EPM/Unifesp), São Paulo, SP, Brasil; 2 Universidade Federal de São Paulo Departamento de Endocrinologia Escola Paulista de Medicina Universidade Federal de São Paulo São Paulo SP Brasil Departamento de Endocrinologia, Escola Paulista de Medicina, Universidade Federal de São Paulo (EPM/Unifesp), São Paulo, SP, Brasil; 3 Universidade Federal de São Paulo Laboratório de Esteroides Escola Paulista de Medicina Universidade Federal de São Paulo São Paulo SP Brasil Laboratório de Esteroides, Escola Paulista de Medicina, Universidade Federal de São Paulo (EPM/Unifesp), São Paulo, SP, Brasil; 4 Universidade Federal de São Paulo Departamento de Medicina Escola Paulista de Medicina Universidade Federal de São Paulo São Paulo SP Brasil Departamento de Medicina, Escola Paulista de Medicina, Universidade Federal de São Paulo (EPM/Unifesp), São Paulo, SP, Brasil

**Keywords:** Quality of life, Turner syndrome, sexual steroids, hypogonadism

## Abstract

**Objectives:**

We aimed to measure the quality of life (QoL) of patients with Turner syndrome (PTS) and determine the extent to which their clinical or laboratory alterations influence QoL compared to reference women (RW) of the same age range.

**Subjects and methods:**

From Dec-2013 to Dec-2014, 90 participants were recruited. They were 18 years and older: 48 with Turner syndrome (TS) (PTS) and 42 without (RW). Recruited subjects completed the Portuguese version of Short Form 36 (SF-36) questionnaire, and blood was drawn to measure LH, FSH, oestradiol (E2), progesterone (P4), SHBG, and SDHEA (by ECLIA) and testosterone (by LC MS/MS).

**Results:**

Age and schooling were similar between groups. The most common occupations for PTS were health worker, administration and education, and health worker or cashier for RW. Most participants were Catholic or Evangelical. Eighty-one percent (39/48) of cases used Hormonal Replacement Therapy (HRT), mostly transdermal (23/39). RW and PTS scored similarly on the SF-36 questionnaire. RW had higher oestradiol (p = 0,01), lower FSH (p = 0,01) and higher testosterone (p = 0,01) than PTS. Concentrations of P4, LH, SHBG or SDHEA were similar. Significant associations were found among QoL and hormones (E2 with Vitality and LH with Physical Role) only in the PTS group.

**Conclusions:**

PTS do not consider that TS affects their QoL as measured by domains on the SF-36. Oestradiol was related with QoL, emphasising the importance of HRT.

## INTRODUCTION

In 1938, Dr. Henry Turner described a group of signs and symptoms that were named after him as Turner syndrome (TS) (1). In 1954, clinicians at the the *Escola Paulista de Medicina* (EPM for short) as reported in Décourt and cols., associated an absence of Barr’s corpuscles in skin biopsy with the clinical features described by Turner (2). In 1959, Ford and cols. described the karyotype, demonstrating altered sexual chromosomes (3). The rate of this genetic condition is ½,000 female newborns, and the most common karyotype in TS is the 45,X (monosomy) condition (60%) (4), though in 99% of cases, the 45,X embryo does not reach full term (5). TS-related genetic anomalies may manifest as clinically observable characteristics such as short stature, low-set ears and hair, webbed neck, genu varum, or syndactilia. Clinical complications may include: gonadal failure, osteoporosis, hearing loss, and sometimes severe cardiovascular diseases (6). Additionally, some psychological problems that can appear include low self-esteem, difficulty with body image and self-consciousness about clinical characteristics (7).

Quality of Life (QoL) is an idea linked to one’s welfare and the capability to live to the fullest. Moreover, concepts like QoL, social functioning, and health conditions are considered synonymous (8). As with any chronic disease, patients need informed, attentive and professional follow-up to maintain a satisfactory QoL (9). Therefore, treatment aims to help the patient reach the best physiological and equilibrated state (10). The “health level” of a person is subjective and can only be verified using standardized clinical procedures, such as measuring vital signs, or by consensus between experts. Thus, guidelines or validated resourceful questionnaires were designed to add objectivity to the measurement of QoL, and the most widely used of these have been the World Health Organization Quality of Life (WHOQoL) instrument (8), and the Short Form 36 Health Survey (SF-36, J.E. Ware, QualityMetrics and Medical Outcomes Trust) (11). The SF-36 has been used extensively in Brazil to assess QoL in rheumatoid arthritis (12) and renal insufficiency (13), having been adapted and translated to Brazilian Portuguese in UNIFESP by Ciconelli and cols. in 1999 (14). Additionally, Naess and cols. (15) previously used the SF-36 in a TS population in Norway.

We hypothesised that patients diagnosed with TS may have QoL impairments and when these patients are clinically stable with normal laboratory results, they may have different specific domain scores on the SF-36 questionnaire compared to reference women. We also examined the influence of sexual steroids on patient QoL.

## SUBJECTS AND METHODS

From Dec-2013 to Dec-2014, 90 participants were recruited, being 48 patients with Turner syndrome (PTS), from an endocrinology outpatient clinic at a Federal University in Sao Paulo, Brazil, and from the Support Group for Women with Turner Syndrome (*Grupo de Apoio* às *Mulheres com Síndrome de Turner*, GAMT). To serve as a reference group, 42 healthy women of similar age and normal weight and height, who could be companions, relatives, or students of Unifesp (Medicine or Nursing schools) or clinic workers were included. The project was approved by Universitary Hospital Sao Paulo, Unifesp Research Ethics Committee (CAAE number 24501813.6.0000.5505), and it complies with the Helsinki Declaration. The enrolled participants signed the informed consent term attached to the study questionnaire. This study also followed all biohazard measures and complied with the local regulations of the Ministry of Health of Brazil MS 466/12 for Research in Human Subjects.

According to data collected in 2010 by the Brazilian Institute of Geography and Statistics (Instituto Brasileiro de Geografia e Estatística, IBGE) (16), Brazil’s population at the time was 190,732,694, while São Paulo city had 5.924.872 women. Considering the TS rate of 1/2,000 female newborns, this means that as many as 2.962 women in the city could have TS, and our studied population is representative of the studied population as in Unifesp Outpatient centerthere were 102 clinical files of girls or women with TS and almost half of them participated. Participants were 18 years or older and were eligible for the study as PTS if they had any karyotype classified as TS. Participants were eligible for participation as Reference Women (RW) if they were healthy and had no exclusion criteria, as are the following: 1) severe illness, 2) currently using birth control medication, 3) had any history of ovarian disease (such as policystic ovary syndrome), or 4) currently pregnant or breastfeeding. Collected information included the presence of TS, age, schooling, occupation classified according to the guidelines of the Brazilian Ministry of Work and Employment (2002) (17), religion, use of hormone replacement therapy (HRT) and the method of administration, and the use of any other medications.

For PTS, clinical and laboratorial data for thyroid function, comorbidities and medicines were collected from the medical records. All patients were asked to bring their audiometry evaluation. Two PTS had been undergone a webbed neck correction plastic surgery.

We chose the SF-36 as our QoL questionnaire because it is an easy, general, and widely used patient self-reported outcome survey (18). The SF-36 contains 36 self-report items assessing 8 health domains (Physical Functioning, Physical Role, Bodily Pain, General Health Perceptions, Vitality, Social Functioning, Emotional Functioning and Mental Health). Each domain receives its own score ranging from 0 = worst QoL to 100 = best QoL. We utilised the Portuguese version of the SF-36 developed by Ciconelli and cols. (14).

Serum concentrations measured included: LH (mlU/mL), FSH (mUl/mL), oestradiol (E2) (pg/mL), progesterone (P4 ng/mL), sex hormone-binding globulin (SHBG nmol/l) and dihydrotestosterone sulphate (DHEA-S µg/dL) by electrochemiluminescence immunoassay (ECLIA) and testosterone by liquid chromatography mass spectrometry (LC MS/MS). Blood samples were collected at follicular stage of menstrual cycle for RW, and follicular-like period for PTS (7-12 days after start of menses). Sera were obtained by centrifugating 7 to 10 mL of blood per patient at 2.000 rpm per 13 to 15 minutes in a SORVALL brand centrifuge. Samples were then frozen and stored at -20 degrees Celsius until processing. Thawing occurred twelve months after the blood draw of the first participant; measurement of all the ECLIA-measured hormones was executed at the Laboratory of Steroids of Unifesp in December 2014. Roche Cobas laboratory reactives were used in an ELECSYS 2010 spectrophotometer. Testosterone was measured in an AB sciex QTrap 5500. In addition to testosterone, all the samples and reactions by ECLIA were processed together and on the same occasion dosed in the Special Chemistry Laboratory of Albert Einstein Hospital; later, testosterone was processed in March 2015. In the case of an undetectable result, the lower limit of detection was used as follows: E2 – 5 pg/mL; P4 – 0,03 ng/mL; LH – 0,1 mUI/mL; FSH – 0,1 mUI/mL; SDHEA – 0,100 mcg/dL; SHBG – 0,350 nmol/L; Testosterone – 10 ng/dL. We excluded too high values.

Results from statistical analyses are presented as medians and absolute or relative frequencies for qualitative or categorical variables, and as means ± standard deviations for quantitative continuous variables. The Kolmogorov Test of Normality was conducted to identify normally distributed variables. The Pearson correlation coefficient was calculated to examine the relationship between two quantitative normally distributed variables and the Spearman quotient for non-parametric quantitative variables. The association between qualitative variables was verified with contingency tables and analysed with the chi-square test. Student’s t-tests were used to identify significant differences between two quantitative normally distributed variables, and the Mann Whitney test was used for non-parametric quantitative variables. ANOVA was used to compare qualitative variables having three or more categories; differences were considered significant if p < 0.05. Analyses were conducted using IBM SPSS Statistics 20 software (New York, United States).

## RESULTS

We analysed 48 PTS and 42 RW. Thirty-seven PTS had the 45,X karyotype (monosomy), two had 45,X/Xip, 4 had 45,X/Xiq, and 5 had other karyotypes. Their final height was 145.6 ± 6.1 cm (from 134 to 158 cm). They were 12.7 ± 9.8 years old when TS diagnose was confirmed (from birth to 53 yo). Twenty (42%) received GH treatment. When we compare the highest (150.5 ± 4.1 cm of height) with the lowest TPS patients (141.0 ± 3.6 cm), divided by the Median, there was no diference in their QoL. However, there was a tendence to Functional Capacity (p 0.065) and General Health Perceptions (p = 0.72) aspects of QoL to be higher in higher TPS patients them in lower ones. Age in the PTS group varied from 18 to 65 years (Median 29.5 years) and from 18-57 years (Median 31.5 years) in the RW group. No significant difference in age between the two groups was observed. Twenty PTS (42%) and fifteen RW (36%) reported having an undergraduate schooling level, and therefore, could have a job and career. Employment domains reported among PTS included education (3/48), business administration (6/48), health (4/48), and food preparation (4/48). Employment areas among RW included health worker (13/42), cashier (4/42) and food preparation (2/42). Whereas two PTS reported that they worked with machines (one in a factory, another in an amusement park), no RW reported that occupation type. In addition, 9 PTS and 11 RW declined to state their religion; however, the majority of the participants was Christian (Catholic, 52% of PTS, 31% of RW, or Evangelical, 18,8% PTS and 28,6% RW).

Thirty-nine PTS (81%) were taking HRT, three were at menopausal age with no HRT, one used to have migraine with any kind of estrogen replacent, and the other 5 were non-adherents. Of the 39, 23 patients (59%) used transdermal administration (patch or gel), and 16 used oral pills (41%), with the cicle starting at day 1 each month, estrogen replacement everyday and progestogen replacement from days 10 to 13 till 20 to 23 every month. Regarding other reported medications in PTS group, 26 (54%) patients took none, considering separately the use of HRT and eleven took more than one. The most common medicines included calcium or vitamin D, used by 15 patients, and levothyroxine, taken by 12 patients. Four patients were taking serotonine reuptake inhibitors, ten hypertensive TPS were taking losartan and/or amlodipine, and 2 diabetics were taking metformin. All had clinical and laboratorial exams well compensated. Thirty-one participants (73%) from the RW group reported taking no medication, five used thyroid hormone replacement, 2 used serotonine reuptake inhibitors, 1 used a blood-thinner (blood clot prevention), 1 used metformin, one was taking losartan plus clortalidone, and the last one used an antidopaminergic medication. Two RW were at menopause age.

Regarding QoL, analyses revealed no difference in scores from SF-36 between both groups (Table 1).

**Table 1:** Scores from the SF-36 for patients with Tuner syndrome and for the reference women

	Patients with Turner Syndrome	Reference women	
	
	n = 48	n = 42	
**DOMAINS**	**Mean ± SD**	**Mean ± SD**	**P**
			
General Health Perceptions	59 ± 17	61 ± 16	0.93 ns
Mental Health	57 ± 20	58 ± 20	0.62 ns
Vitality	60 ± 22	62 ± 21	0.23 ns
			
**DOMAINS**	**Median (min-max)**	**Median (min-max)**	**P**
			
Physical function	85 (10-100)	87,5 (35-100)	0.73 ns
Physical role	100 (0-100)	100 (0-100)	0.63 ns
Social role	75 (0-100)	75 (0-100)	0.72 ns
Emotional role	100 (0-100)	100 (0-100)	0.60 ns
Bodily pain	84 (0-100)	73 (10-100)	0.34 ns

Scores from the SF-36 do not have a unit of measurement.

Twenty-seven percent (13 of 48) of TS had hypoacusia, though only one had severe hypoacusia, and that was approprietadly corrected with an earpiece. When we compared QoL data, the TS patients with hypoacusia did not differ with normal hearing TS nor with RW.

Blood samples were not collected in two PTS and three RW (because of certain difficulties returning to the facility); therefore, those individuals were excluded from laboratory analyses*,* leaving a total of 45 PTS and 40 RW. One RW presented a level of oestradiol of 1,157 pg/mL, and thus was excluded because it exceeded limits (12.5 to 166 pg/mL in the follicular phase). Her other hormonal levels were normal. In seven PTS and one RW, the result was undetectable and rounded up to the minimum value in the detection range for oestradiol levels. The same was done for LH levels in one PTS and one RW, and for testosterone levels in 17 PTS and two RW with undetectable results. Table 2 presents hormone results for the study.

**Table 2:** Laboratory results for Patients with Tuner syndrome and the reference women

	Turner Women		Reference Women		p	Laboratory Reference
	
	Mean ± SD	Range (min/max)	Mean ± SD	Range (min/max)		
E2 (pg/mL)	50.1 ± 67.2	5.0-357.7	105.6 ± 91.5	5.0-398.8	0.01	12.5-166
P4 (ng/mL)	1.1 ± 0.4	0.1-21.7	2.5 ± 4.9	0.2-19.6	0.3	0.2-1.5
LH (mIU/mL)	26.6 ± 20.5	0.1-99.1	41.3 ± 167.4	0.1-1066	0.56	2.4-12.6
FSH (mIU/mL)	65.6 ± 46.5	15.2-216.7	11 ± 21.7	0.4-135.1	0.01	3.5-12.5
SDHEA (µg/dL)	189.5 ± 111.3	21.7-498.7	195.5 ± 108.5	42.1-449	0.10	33.9-337 (age 10-44)
SHBG (nmol/L)	65.6 ± 46.5	15.2-216.7	84 ± 56.6	18.2-282.6	0.8	32.4-128 (age 20-44)
T (ng/dL)	12	10-35	23	10-67	0.01	10-38*

E2: oestradiol; P4: progesterone; LH: luteinizing hormone; FSH: follicle stimulant hormone; SDHEA: sulphate-dehydriepiandrosterone; SHBG: sex hormone-binding globulin; T: total testosterone; pg/mL: picograms per mililitre; ng/mL: nanograms per millilitre; mUI/ml: mili international units per millilitre; μg/dL: micrograms per decilitre; nmol/L: nanomol per litre; ng/dL: nanograms per decilitre. * Lab Reference for Tanner stage 5.

Levels of oestradiol and testosterone were significantly higher in RW than PTS, and FSH was higher in PTS (all p < 0.01). The observed correlations for the PTS group between hormone concentrations and QoL were only with E2 and Vitality (r_S_ = 0.32, p = 0.04) and LH with Physical Role (r_S_ = 0.33, p = 0.02). None such correlations were found in the RW group.

## DISCUSSION

In our study, QoL was similar between both groups when measured using the SF-36 questionnaire. Additionally, we observed no differences in education, occupation or religion. However, we noticed that even when PTS under HRT had lower levels of oestradiol than controls, they were similar to those of a normal woman in her follicular stage according to the Unifesp laboratory reference ranges. The levels of oestradiol correlated with the Vitality domain of the SF-36 only in PTS, showing that levels of oestradiol may affect QoL if lower than reference range.

Schooling is not impaired in the TS group according to our study, showing that a diagnosis of TS was not influential on patients’ education level or on the possibility of patients completing their studies or becoming professionals. This finding was supported further by the observation that people in the PTS and RW groups had similar careers and occupations. This is consistent with findings from Naess and cols. on education, but they found a difference on work areas (more PTS in education, more RW in Economics or Administration) (15) that we did not, probably due to characteristics of the educational system. Approximately 50% of our participants had an undergraduate or graduate level of education, and 65% of the participants in the Norwegian study had achieved 15 years of schooling, fostering the observation of the difference in the placement of PTS. By the other side, Stocholm et al found in Denmark that even if the patients with Turner syndrome and their controls had no difference in the possibility to get a bachelor´s degree, they did have difference in their income, until the third decade (19). Unfortunately, we did not ask for the income in the present study.

The religious affiliations reported by the participants are common in Brazil (20), and there was no significant difference in declared religions between the groups. The feelings of faith and trust in “something or someone” that can provide material and spiritual protection and love doubtlessly influence individual feelings of welfare (21). Religious practice is related to quicker and better recovery in people with physical or mental ailments, and with the general sensation of health and longevity (22). Some mental issues related to TS can be “made up for” or “caught up” with age and appropriate environment stimuli (23).

Differently from the Norwegian study of Naess and cols. (15), we noticed that only on some of SF-36 domains, in particular scores for General Health Perceptions and Mental Health, our participants’ scores were lower in the PTS than in the RW, though no significance was found. The differences in cultural and social characteristics of two different countries should be considered, as they can affect people’s opinions about QoL. TS-specific QoL has also been measured in Israel by Zucherman-Levin and cols (with the WHO-QoL), who showed that treatment with androgens improved general health, mood, feelings of well-being, sexual desire and relationships (24).

Ciconelli and cols.’s investigating QoL in patients with rheumatoid arthritis (RA), found lower scores in QoL regarding characteristics specific to RA, such as Physical Function, Physical Role, Emotional Role and Bodily Pain or higher scores for General Health Perceptions, Social Role, Mental Health and Vitality (16). However, the observable characteristics of TS, such as webbed neck or facial stigmata, did not influence the QoL in our patients, though, the shortest PTS tended to have lower scores for domains Functional Capacity and General Healthy Perceptions. This pinpoints the importance of earlier diagnose of TS and GH taking for a greater period, improving final height to these patients and possibly their QoL.

In addition to HRT, patients could be using other kinds of medication for eventual comorbidities, such as hypertension, diabetes mellitus, dyslipidaemia or depression. In our study, the PTS received more medications than the RW, independently of HRT. Therapy findings showed 19% of the PTS were not taking HRT, either because of noncompliance or appropriate age for menopause, meaning the PTS’s reasons for noncompliance need to be addressed in the medical consult. Deverney et al described on their study that the compliance to medical care is influenced by type of physician, paternal socioeconomic class, education level, number of Turner syndrome disease components, size of the medical center following the patient in childhood, and physical health dimensions of Short Form 36 questionnaire (25). On a Dutch study by Freriks and cols., 30% of their sample had not accurate follow-up, and 15% were not receiving HRT at the time (26).

In the present work, the laboratory findings showed that PTS can obtain laboratory results quite similar to those of women in their follicular phase, though mean levels of oestradiol were lower. Additionally, the correlation between E2 and the Vitality domain of SF36 questionnaire in PTS combined with the lack of such a relationship in RW is attributed to a manifestation of subclinical hypoestrogenism, present in PTS but absent in the eugonadal RW. Suggesting that vitality decreases without HRT would be an argument in favour of compliance with it. A correlation was not found in any group between a domain of QoL and P4, but RW had a wider range of results because they have natural cycles. The reason for this finding is probably related to the duration of the progesterone pills in the scheme the PTS use (recommended for 10 to 14 days during the month) (26), while in women with normal cycles, the levels will tend to vary according to the phase they are in, and the changes in concentration influence one’s feelings of welfare (27). The fact of PTS taking a progestogen for short periods during the month (from days 13 to 23 every month) can be related to the non-association of this hormone with QoL

FSH levels but not those of LH were significantly different between groups (FSH higher in PTS, achieving levels of menopause). The effects of gonadotrophins in the absence of gonads were described in the classic study of Thales Martins in 1931 (28), and FSH does not diminish with HRT because of the lack of FSH receptors in the dysgenetic gonad (29). In addition, Healy and cols. suggested the need for a counterregulation factor secreted by the gonads such as inhibin, to keep FSH at normal levels (18). Inhibin in women is produced in the granulosa cells of follicles. In the study by Gravholt and cols., levels of inhibin-A and inhibin-B in Turner women were generally undetectable. However, some of them showed slightly lower levels of gonadotrophins and detectable inhibin-B (at prepubertal levels), showing that the dysgenetic gonad of PTS is not sufficiently able to participate in the counterregulation of FSH due to the early and accelerated destruction of follicles before birth (30,31).

SHBG levels were not significantly different between the PTS and RW groups in our study. However, Hampl and cols., in their study in the Czech Republic, reported that untreated PTS had low SHBG, while PTS receiving GH had even lower concentrations, yet PTS under oestrogens and HRT had increased levels (32). On one hand, SHBG is considered as a marker of insulin resistance (33), on the other, Turner patients have greater insulin resistance, Teoretically the counterbalance between the expected increase of SHBG with HRT and its decrease amidst insulin resistance can justify the absence of difference in SHBG levels between PTS and RW in the present study.

We observed in our patients that testosterone levels were lower in PTS than in RW, and many PTS had concentrations below the detection limit of method, which was 10 ng/dl. However, the maximum levels found in PTS were in the range of women in Tanner stage 5 of puberty. Tanner stage was not assessed because participants were 18 or older, presumably done with their pubertal development, so it can be assumed that they were M4P4 or more developed. Testosterone levels were not associated with any domains measured by SF-36, probably because the questions did not include inquiries about sexual desire/performance, feelings of aggressiveness or weight changes. Even though testosterone has been found by immunohistochemical methods in hilium cells of PTS gonads, obtained after prophylactic gonadectomy, it is possible that these levels are too low to be found in blood (34).

Though our sample was representative of ST population, the fact that only women who willingly presented to the clinic participated may cause some bias of selection in the sample, and is a limitation of our study. Also, this study lacks sufficient data to describe the situation of untreated PTS, as the population without treatment was quite small (9 patients).

In conclusion, the women with TS did not report negative impacts of the diagnosis on their QoL as measured by the SF-36 questionnaire. Patients with TS adapt themselves well to the challenges of their condition and to factors that affect QoL of patients undergoing treatment with HRT, which are the same in people without TS. We suggest that psychological support be provided in a model based on that genetic characteristic. Furthermore, treatment with HRT is able to equate the QoL of people with or without TS, and the hormonal profile was similar to the profile of a normal woman in her follicular phase. In the age range of the participants in our study, the desirable amount of naïve or untreated people, or patients without follow-up should be low because TS is present at birth. Therefore, because oestradiol was associated with vitality, it shows the importance of HRT, and the association of LH with physical aspects measured by the SF-36 corroborates this statement. Moreover, the absence of effect of HRT on FSH is due to the absence of gonadotrophin feedback from inhibin. Finally, though very low testosterone concentrations are related to underdeveloped or streak gonads, there is no association between low testosterone concentrations and QoL in our patients.
